# Supplementary figures and images for: Patient-derived iPSCs link elevated mitochondrial respiratory complex I function to osteosarcoma in Rothmund-Thomson syndrome
Source: PLoS Genet. 2021 Dec 29;17(12):e1009971. doi: 10.1371/journal.pgen.1009971 (PMC8716051; doi:10.1371/journal.pgen.1009971)

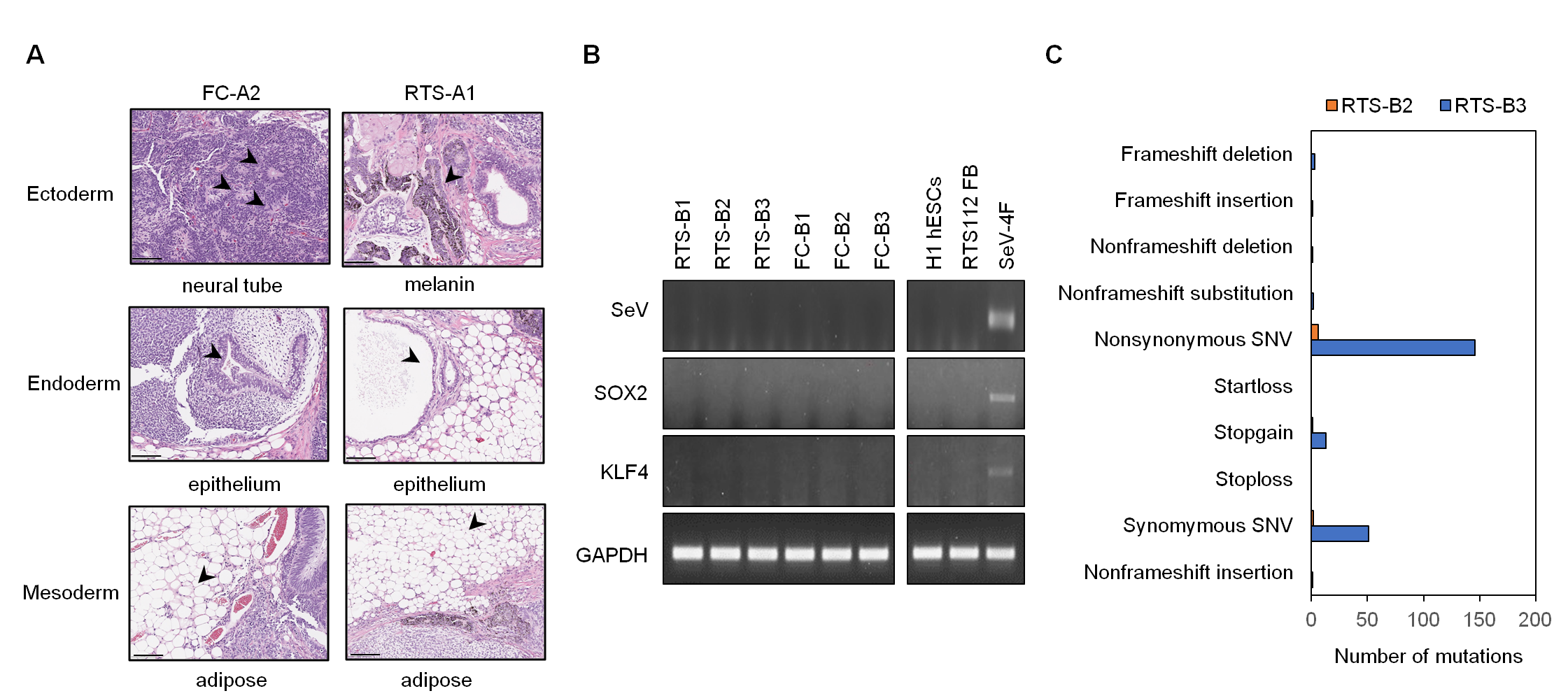

Supplement: S1 Fig — (A) In vivo teratoma formation assay demonstrates three germ-layer differentiation abilities of RTS and FC iPSCs. Scale bar, 200 μm. (B) PCR detection of SeV genome and transgenes indicates that RTS and FC iPSCs are footprint-free. (C) Somatic variant analysis of RTS-B2 and RTS-B3 iPSCs suggests that limited somatic mutations are generated during cellular reprogramming. (TIF) [file pgen.1009971.s001.tif]

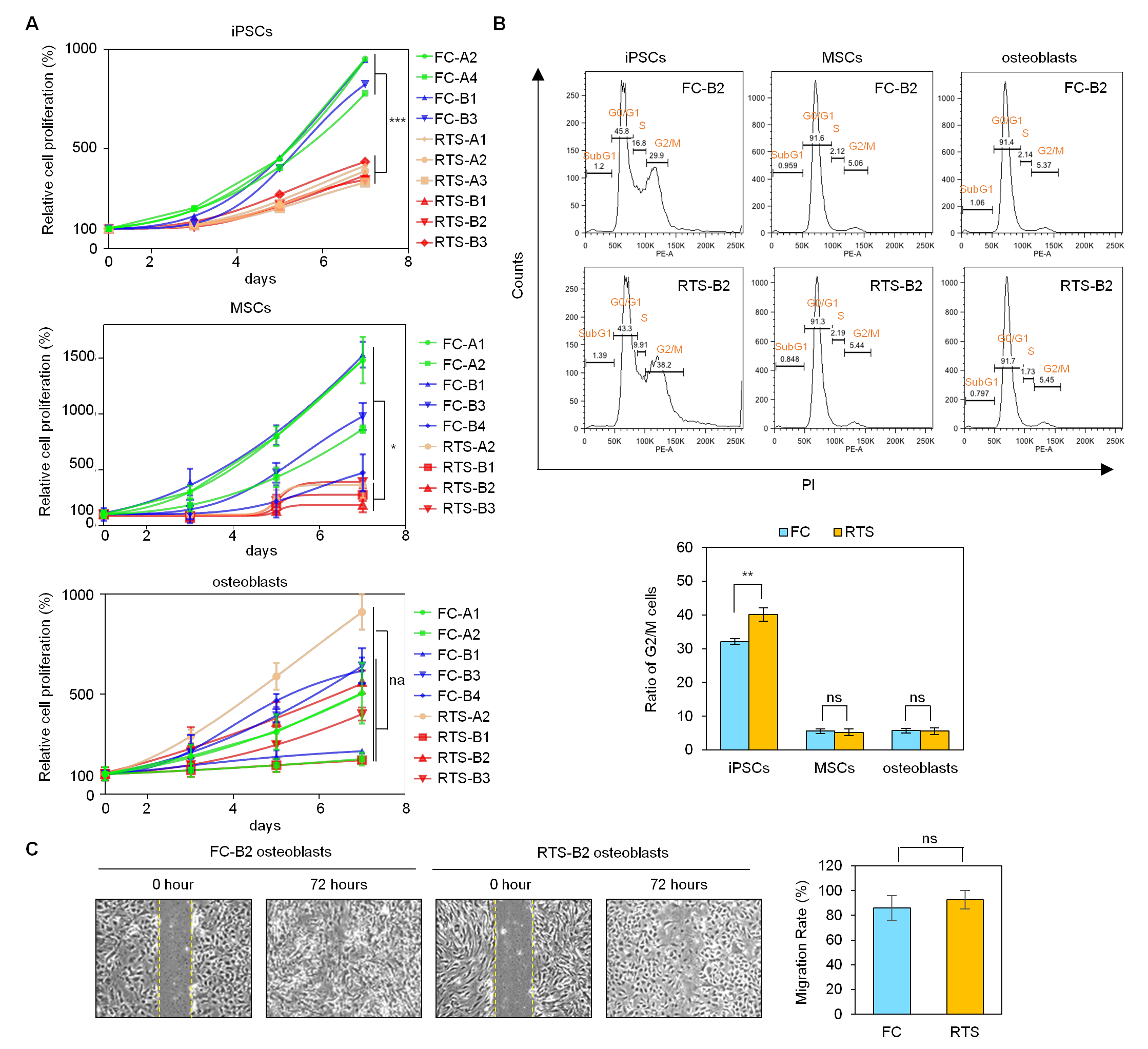

Supplement: S2 Fig — (A) Relative cell proliferation of RTS and FC iPSCs (upper panel), MSCs (middle panel), and osteoblasts (lower panel). n = 3 biological replicates. (B) Cell cycle profiles of RTS and FC iPSCs, MSCs, and osteoblasts are performed using PI staining by flow cytometry. (Upper) Cell cycle profiles of RTS iPSCs, MSCs, and osteoblasts. (Lower) Bar histogram shows the percentage of cells in the sub-G1, G0/G1, S, and G2/M phases. n = 3 biological replicates. (C) Wound healing assay determines the cell migration ability of RTS and FC osteoblasts. Wound healing assays were performed at 72 hours in RTS and FC osteoblasts. (Left) Representative phase-contrast microscope images show the area covered by RTS and FC osteoblasts at 72 hours after wounding. (Right) The cell migration rate is determined by the rate of cells moving towards the scratched area upon time measured. n = 3 biological replicates. (TIF) [file pgen.1009971.s002.tif]

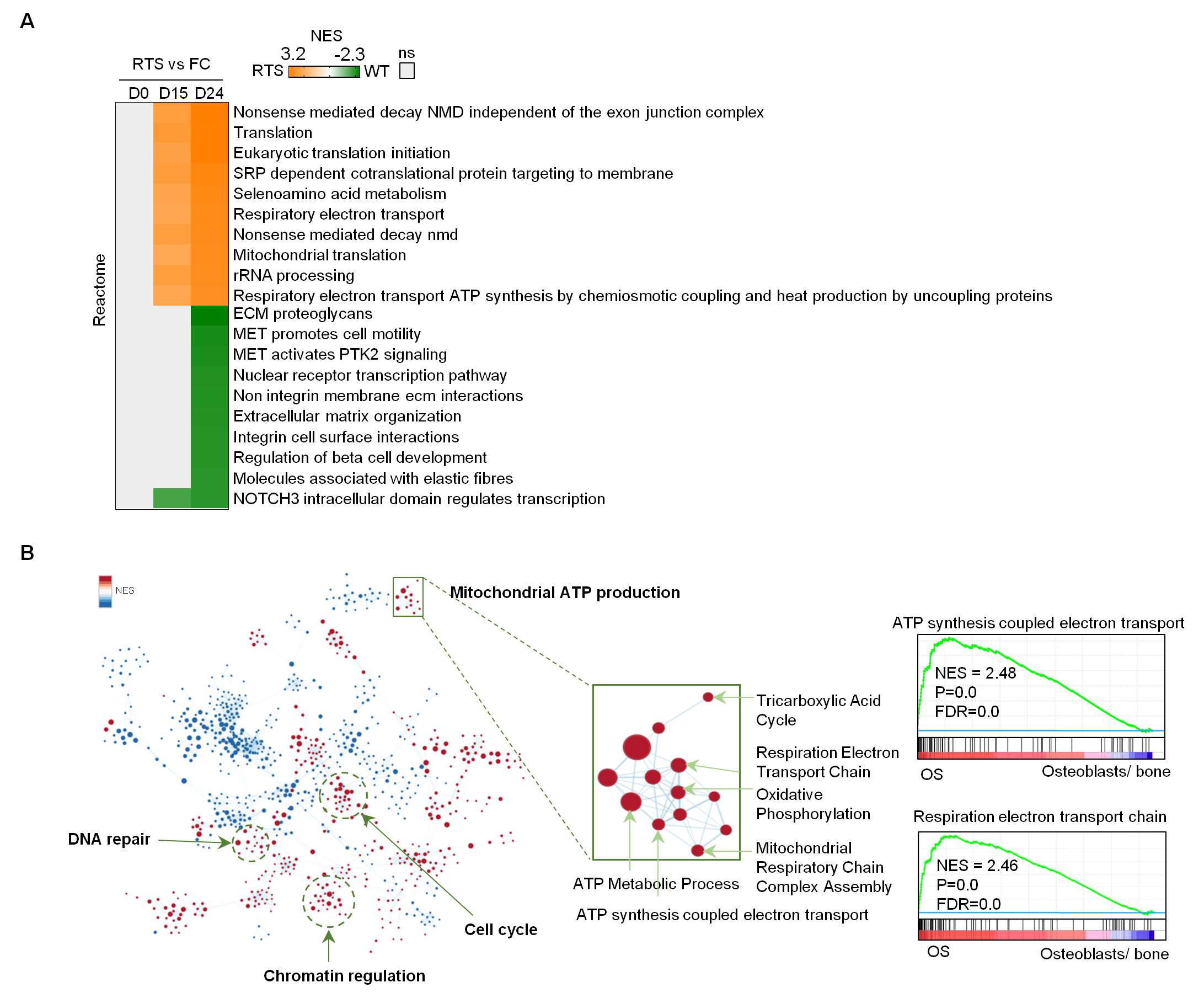

Supplement: S3 Fig — (A) Reactome analysis demonstrates enrichment of multiple canonical pathways in RTS compared to FC MSCs (D0), pre-osteoblasts (D15), and osteoblasts (D24). (B) Left, GO_BPs enriched in osteosarcoma by EnrichmentMap analysis. Network visualization of gene sets enriched in osteosarcoma compared with normal bone tissues and osteoblasts (p value <0.05, FDR q value <0.1) indicates that GO_BPs involved in DNA repair, chromatin regulation, cell cycle, and mitochondrial ATP production and repair are enriched in osteosarcoma. The number of enriched genes in each GO is displayed as node size, with closer distance between nodes representing increased overlap between genes in ontologies. Enriched gene sets in osteosarcoma are displayed in red and enriched gene sets in bone tissues and osteoblasts in blue. Right, representative enriched gene sets (ATP synthesis coupled electron transport and respiration electron transport chain) involved in mitochondrial ATP production are shown. (TIF) [file pgen.1009971.s003.tif]

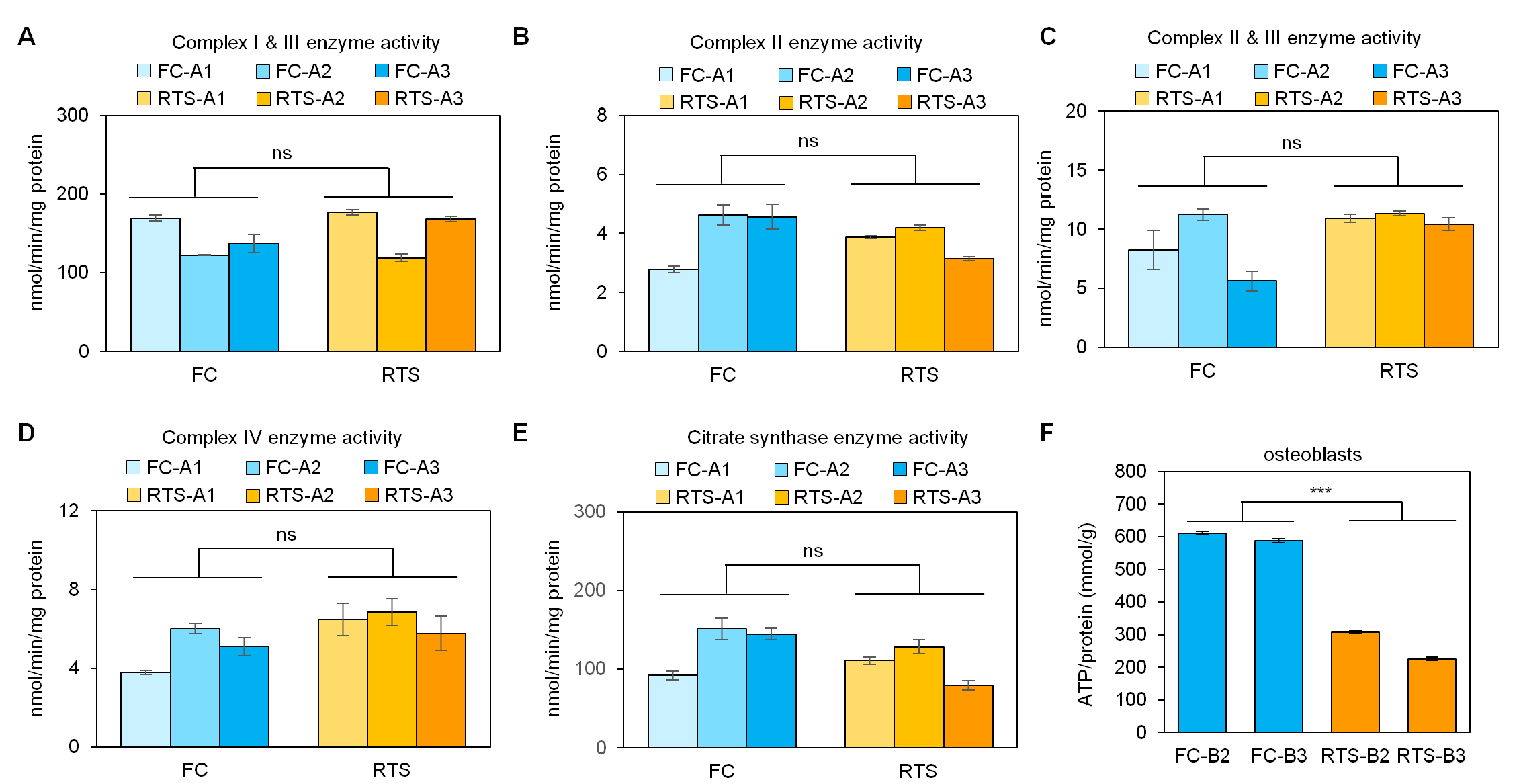

Supplement: S4 Fig — (A-E) Enzyme activity of complexes I and III (A), complex II (B), complexes II and III (C), complex IV (D), and citrate synthase (E) show no significant difference between RTS and FC osteoblasts, leaving complex I as the only component of the electron transport chain with upregulated activity. n = 3 biological replicates. (F) The decrease of total ATP levels is observed in RTS osteoblasts compared to FC osteoblasts. n = 4 biological replicates. (TIF) [file pgen.1009971.s004.tif]

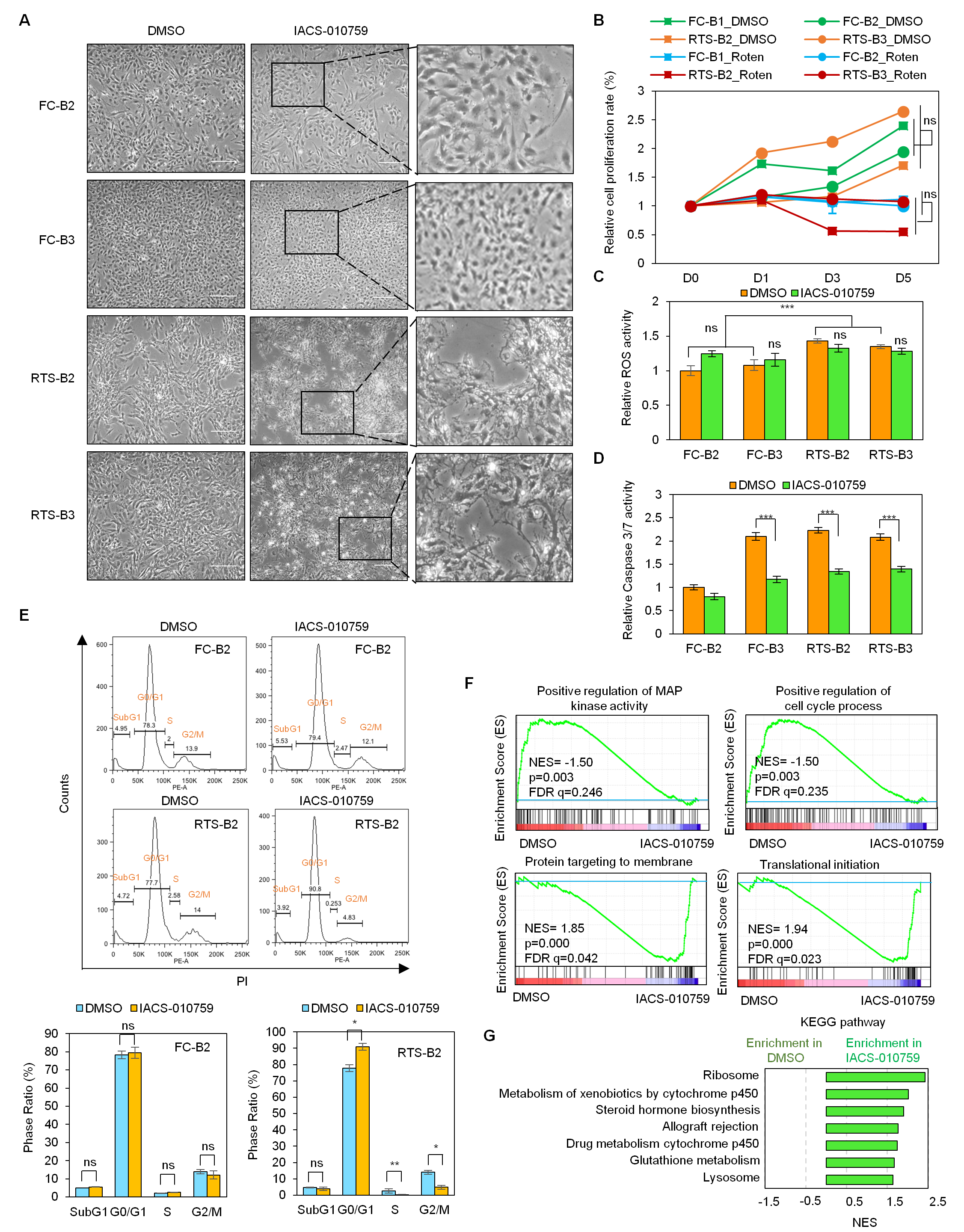

Supplement: S5 Fig — (A) IACS-010759 alters RTS osteoblast cell morphology from a spindle shape to an irregular shape. Scale bar, 100 μm. (B) Cell proliferation assay indicates that Rotenone impairs cell proliferation of RTS and FC osteoblasts. n = 4 biological replicates. (C) ROS activity assay demonstrates that IACS-010759 does not induce ROS production in both RTS and FC osteoblasts. n = 3 biological replicates. (D) Cell apoptosis assay indicates a decrease of Caspase 3/7 activities upon IACS-010759 treatment in both RTS and FC osteoblasts. n = 3 biological replicates. (E) Cell cycle profiles of RTS and FC osteoblasts are performed upon IACS-010759 treatment. (Upper) Cell cycle profiles of IACS-010759-treated RTS and FC osteoblasts. (Lower) Bar histogram shows the percentage of cells in the sub-G1, G0/G1, S, and G2/M phases. n = 3 biological replicates. (F) Representative GO_BPs influenced by IACS-010759, including protein targeting to membrane, translational initiation, positive regulation of MAPK activity, and positive regulation of cell cycle process. (G) KEGG pathway analysis confirms upregulation of ribosome function in IACS-010759-treated RTS osteoblasts. (TIF) [file pgen.1009971.s005.tif]
