# Supplementary material for: Patient-derived iPSCs link elevated mitochondrial respiratory complex I function to osteosarcoma in Rothmund-Thomson syndrome
Source: PLoS Genet. 2021 Dec 29;17(12):e1009971. doi: 10.1371/journal.pgen.1009971 (PMC8716051; doi:10.1371/journal.pgen.1009971)
Supplement: Attachment — Submitted filename: PLoS Genetics Rebuttal Letter_Final.docx [file pgen.1009971.s007.docx]

**Point-by-point response to reviewers’ and editorial comments ((PGENETICS-D-21-00811)**

We thank all the reviewers for their positive assessment of our work and their highly constructive comments. This has helped us design new experiments and revise the manuscript to greatly improve the clarity and the impact of our findings on the field. Based on the reviewers’ comments, we performed new experiments, included 20 new data sets (Figs 1C, 1D, 3F, 5C, 5D, 5E, 5H, 5I, 5J, S1C, S2A, S2B, S2C, S4F, S5A, S5B, S5C, S5D, and S5E as well as Table S1), and revised the manuscript accordingly. For the reviewers’ convenience, we have made edits in the revised manuscript and highlighted the major changes. We trust that all comments have been addressed satisfactorily either through additional experiments or through better explanation to clarify specific points.

**Reviewer #1**:

1. *The authors do not provide any evidence that the RECQL4 mutations are present in the iPSC cells derived from the patients - whilst it is assumed (and I think this assumption is reasonable) the mutations should be sequenced and provided from the iPSC. The authors also should provide an assessment, and if possible a western, to understand if the mutation generates a predicted protein product and if so how and where does it localise. Ideally using the endogenous protein but otherwise with mutations in a cDNA.*

**Response:** We appreciate the reviewer’s comments. **First**, we performed Sanger sequencing to confirm the indicated RECQL4 pathogenic variants in RTS-A and RTS-B fibroblasts (Fig 1C of the revised manuscript). The RECQL4 (c. 2719C>T/Q907X; [=]) mutation in RTS-A iPSCs and RECQL4 (c.[1568G>C;1573delT];[IVS11+32del24]) mutation in RTS-B iPSCs were further confirmed by Sanger sequencing and whole exome sequencing (See below), respectively. To avoid redundancy, we only show iPSC sequence results here for your reference. **Second**, immunoblotting showed that RTS-A fibroblasts present the loss of RECQL4 proteins and RTS-B fibroblasts contain truncated RECQL4 proteins (Fig 1D of the revised manuscript). These findings indicated the loss of RECQL4 function in RTS-A and RTS-B patients. In addition, due to the lack of IF-qualified RECQL4 antibody to detect endogenous RECQL4 protein properly, we cannot determine the subcellular localization of truncated RECQL4 proteins in RTS-B cells.

RTS-A1 iPSCs-Sanger sequencing

RTS-B fibroblasts and RTS-B2/3 iPSCs-WES

(please see the next page)

**2.** *Do you know the mutational burden of the RTS patient derived cells to be able to conclude that the observed phenotypes are solely due to RECQL4 mutations? Exome sequencing would resolve this.*

**Response:** We appreciate the reviewer’s comments on the mutational burden of RTS iPSCs. Based on previous studies, it is uncommon to create a significant mutational burden during reprogramming, suggesting the safety of iPSC generation using Yamanaka four factors. Also, we applied multiple iPSC-derived osteoblasts to study RTS pathological mechanisms caused by RECQL4 pathogenic variants. It is unlikely that all RTS iPSCs and iPSC-derived osteoblasts contain specific mutations which lead to oncogenic transformation. To further clarify this question, we performed a whole exome sequencing (WES) of RTS-B fibroblasts and their corresponding iPSCs (RTS-B2 and RTS-B3). In agreement with the previous studies, few somatic mutations were generated during cellular reprograming (Fig S1C and Table S1 of the revised manuscript). The number of somatic variants resembles the level of mutations found in reprogrammed iPSCs as reported previously (Rouhani FJ et al., PLoS Genet. 2016 Apr 7;12(4):e1005932). It is worthy to mention that many reprogramming-associated mutations actually pre-existed in fibroblasts subpopulations at low frequencies (Gore A et al., Nature. 2011 Mar 3;471(7336):63-7). No additional mutations related to known tumor suppressor genes and oncogenes (e.g., p53, RB1, and RAS) were detected in RTS-B iPSCs compared to RTS-B fibroblasts. Therefore, the RTS phenotype and its underlying pathological mechanisms (e.g., elevated mitochondrial respiratory complex I genes and function) are indeed caused by RECQL4 pathogenic variants rather than other gene mutations.

**3.** *One of the major advantages of patient derived iPSC is the ability to use human cells directly to understand the biology. It also offers the powerful experimental model of generating gene corrected isogenic cell lines that can be used to allow the most rigorous and robust comparison possible. It is not clear to me why the authors have not attempted to use RECQL4 corrected isogenic cell lines to undertake the analysis in place of, or at least alongside, those derived from a heterozygous parent. The analysis of isogenic pairs of cells would potentially discern subtle biology and also control for all other mutations that may be present in any individuals sample. Whilst the parent derived cells are a control, they are derived from a significantly different age of human, have had different exposures and responses to exposures for life.*

**Response:** Thanks for the reviewer’s comments. We agree that the generation of isogenic pair of cells would be ideal to allow the most rigorous and robust comparison. However, due to the unusual and complicated RECQL4 genome structures (21 exons and 20 introns in 6kb) (J Natl Cancer Inst. 2003 May 7;95(9):669-74; please see the below figure), we encountered difficulties to apply proper correction donor vectors and CRISPR/Cas9 (or TALENs) to perform genome editing on RECQL4 genome regions without affecting *RECQL4* mRNA splicing when we initiated this project. In addition, many RTS-associated RECQL4 pathogenic variants are located in intron and splicing junctions, indicating that impaired RECQL4 mRNA splicing caused by mutations leads to RECQL4’s loss of function in RTS patients. To prevent editing-caused RECQL4 function impairment, we decided to apply multiple RTS patient fibroblasts to generate multiple RTS and FC lines to dissect pathological alterations caused by RECQL4 pathogenic variants in RTS patients. A similar approach has been successfully applied in many iPSC studies including ours (Proc Natl Acad Sci U S A. 2018 Nov 20;115(47):E11128-E11137; Cell. 2015 Apr 9;161(2):240-54; Cell Rep. 2015 Oct 20;13(3):504-515; Nature. 2010 Jun 10;465(7299):808-12).

**4.** *Cells proliferating in soft agar are a poor substitute for in vivo tumor assays (text associated with this observation could be tempered in my opinion). I would encourage the authors to either take the osteoblasts and transplant in nude mice or NSG in matrigel and demonstrate that they are tumorigenic.*

**Response:** We sincerely appreciated the reviewer’s suggestion. In fact, we have performed a xenograft study to examine if RTS osteoblasts are capable to grow in NSG mice. Unlike Li-Fraumeni syndrome (LFS) osteoblasts composing hyperproliferative ability *in vivo* (Proc Natl Acad Sci U S A. 2018 Nov 20;115(47):E11128-E11137; Cell. 2015 Apr 9;161(2):240-54), RTS osteoblasts/matrigel were not able to engraft a tumor (or grow) in NSG mice after 3-month transplantation. However, we don’t rule out the possibility RTS osteoblasts can still present *in vivo* tumorigenic ability but more time is needed. Further study with properly optimized substances (e.g., specialized hydrogel to support pre-malignant RTS osteoblasts growth) will be necessary to be applied to characterize whether RTS osteoblasts compose *in vivo* tumorigenic abilities or not.

**5.** *page 10; lines 210-213 - the text states that sporadic osteosarcoma cell lines have increased ATP production pathways as well. Does this imply that it is a feature of osteosarcoma per se rather than reflective of RECQL4 biology? It is known that loss or mutations in p53, Terc and Rb cause metabolic reprogramming in cells and mice potentially pointing to a more generic feature of tumor suppressors. This is more a comment that a request to do any experiments but may be worth considering.*

**Response:** Thanks for the reviewer’s insightful comments. The text states that “sporadic osteosarcoma cell lines have increased ATP production pathways as well” indicating that increased mitochondrial ATP production could be a common oncogenic mechanism involved in osteosarcomagenesis. Here, we only concluded that increased mitochondrial ATP production pathway (especially, mitochondrial respiratory complex I gene and function) is one outcome and potential pathological mechanism involved in RTS patient associated osteosarcoma. Since metabolic reprogramming is a hallmark of cancer, other osteosarcoma-associated mutations (p53, RB1, and TERC) could also hijack metabolic pathways to promote tumorigenesis. We don’t rule out the possibilities that (1) elevated OXOPHOS in RTS cells is through the alteration of p53, RB1, and TERC signaling; (2) p53, RB1, and TERC mutation also influence OXOPHOS and mitochondrial complex I gene expression in somatic osteosarcomas. Furthermore, we applied GSEA to examine the transcriptome alterations in LFS iPSC-derived MSCs and osteoblasts (Cell. 2015 Apr 9;161(2):240-54), we did not find upregulated OXOPHOS and its related biological processes by GSEA, which excluded the involvement of OXOPHOS in osteosarcoma initiation in LFS patients. However, future studies are required to validate these hypotheses.

**6.** *Previous work has assessed energy metabolism in murine Recql4 mutant bearing cells (PMID: 33361189). This publication did not report and difference in metabolic activity. Do you think this reflects cell type differences, species differences or something else. Given the broad spectrum of clinical manifestations and that these affect cell lineages derived from different germ layers can the authors speculate as to why there is only a discernable difference following osteoblastic differentiation and not one that is apparent in the iPSC or MSC?*

**Response**: Thanks for the reviewer’s comment on these fundamentals of cancer biology. **First,** since Dr. Walkey’s Recql4 study did not report the difference in metabolic activity, we suspect that it could reflect species differences. Also, in the *in vivo* condition, the elevated mitochondrial respiratory complex I activity in osteoblastic lineages could be influenced and masked by paracrine signaling produced by other cell types (osteoclasts and hematopoietic cells) in the bone microenvironment. Therefore, to simplify the complexity of model systems (e.g. mouse and zebrafish), the iPSC platform can serve as an alternative simple human model to re-examine disease etiology. **Second**, we suspect lineage-specific transcription factors (e.g. RUNX2) and signal molecules (Wnt and BMP) are important direct and/or indirect factors to trigger elevated mitochondrial respiratory complex I genes in osteoblasts but not in iPSCs and MSCs. Further studies of cross-talk between RECQL4 and osteoblast signaling are required to elucidate the molecular mechanisms of elevated mitochondrial complex I genes in RTS osteoblasts.

**7.** *At a conceptual level, can the authors discuss further if they think the inhibitor IACS-010759 would be applicable for treating osteosarcoma in patients with germ-line mutations in RECQL4 (such as the RTS patients) where multiple cell types in the body may be affected by exposure to this agent as all cells have the mutation in RECQL4?*

**Response:** Thank you for the comment on the safety of IACS-010759 to be applied to treat RTS patients. As shown in our transcriptome analysis (Fig 3A), enriched mitochondrial respiratory complex I gene expression existed in osteoblasts but not MSCs, implying the unique upregulated mitochondrial respiratory complex I function in RTS osteoblasts but not in non-osteoblastic lineages (e.g., MSCs, etc.). Although we have not examined multiple cell lineages (e.g., neural cells), we suspect that elevated mitochondrial respiratory complex I gene expression and function could be the unique characteristics of osteoblastic lineage cells including osteoblasts and osteosarcoma. Therefore, IACS-010759 may not have an unexpected side effect on non-osteoblastic lineage cells but further studies and comparison of diverse cell lineages’ transcriptome profiles are required to clarify this issue.

**8.** *Demonstrate that the change in MAPK pathway (Fig 5f) is reflected at the protein level and not just from transcriptome signatures.*

**Response:** Based on the reviewer’s suggestion, we performed immunoblotting to confirm the downregulation of EREG (cell cycle), STK39 (MAPK signaling), and phospho-ERK1/2 level in IACS-010759-treated RTS osteoblasts (Figs 5H and 5I of the revised manuscript).

**9.** *Provide additional baseline characteristics of the populations and cells, for example proliferation rates of iPSC, MSC and osteoblasts, cell cycle profiles of the osteoblastic populations and following treatment, evidence of any DNA damage response being active in the cells from MSC to osteogenic cells.*

**Response:** We appreciate the reviewer’s suggestions. **First**, we characterized cell proliferation and cell cycle profile of RTS and FC iPSCs, MSCs, and osteoblasts, and summarized our findings in the revised manuscript (Figs S2A and S2B of the revised manuscript; line 182-193). RTS iPSCs and MSCs have decreased cell proliferation ability compared to FC counterparts, indicating the essential role of RECQL4 in maintaining cell growth (Fig S2A of the revised manuscript, upper and middle panels). However, there was no significant growth difference between RTS and FC osteoblasts (Fig S2A of the revised manuscript, lower panel), implying potential pro-oncogenic transformations (e.g. increased cell growth ability) in RTS osteoblasts. **Second**, we examined the cell cycle profile of RTS and FC osteoblasts upon IACS-010759 treatment and observed IACS-010759 selectively induces cellular senescence of RTS osteoblasts (Fig 5E of the revised manuscript) and altered the cell cycle profile of RTS osteoblasts by increasing G0/G1 and decreasing S and G2/M cell populations (Fig S5E of the revised version). **Third**, we examined γH2AX levels in RTS MSCs and osteoblasts and observed there is no significant change of DNA damage during osteogenesis in RTS cells (please see the figure below).

**Reviewer #2**:

**1.** *Figure 2F: its not sufficient to show the oncogenic potential just by colony formation assay. Authors should try other approach like in vitro metastatic assay to show the oncogenic property of patient derived osteoblasts.*

**Response:** Thanks for the reviewer’s comment. In addition to anchorage-independent growth assay (Fig 2F), we examined if RTS osteoblasts present *in vitro* metastatic potential by the wound healing assay. As shown in Fig S2C of the revised manuscript, there is no significant difference of migration ability between RTS and FC osteoblasts, which may reflect the clinical observation that RTS patient osteosarcomas do not have a prevalence of metastases (J Clin Oncol. 2007 Feb 1;25(4):370-5). Also, we compared cell proliferation among RTS and FC in different stages (iPSCs, MSCs, and osteoblasts), RTS iPSCs and MSCs have decreased cell proliferation ability compared to FC counterparts, indicating the essential role of RECQL4 in maintaining cell growth (Fig S2A of the revised manuscript, upper and middle panels). However, there was no significant growth difference between RTS and FC osteoblasts (Fig S2A of the revised manuscript, lower panel), implying potential pro-oncogenic transformations (e.g. increased cell growth ability) in RTS osteoblasts.

**2.** *Figure 4 A: showed increase in complex I activity. It is important to show the complex I protein level in RTS patients. It would be interesting to check how OXPHOS assembly is affected in RTS patients cell lines.*

**Response:** Based on the reviewer’s suggestion, we have examined the expression of NDUFA7 and NDUFS8 expression by immunoblotting. Consistent with qRT-PCR result, higher expressions of NDUFA7 and NDUFS8 proteins were detected in RTS osteoblasts compared to FC osteoblasts (Fig 3F of the revised manuscript). Also, we appreciate the reviewer’s suggestion to check OXPHOS assembly in RTS cells. Since it requires more mechanistic studies by systematic approaches in order to gain the big picture, it is slightly beyond the focus of the current study and we will explore it in the coming future.

**3.** *Figure 4 E: Decreased ATP levels in RTS patient fibroblasts has been reported previously (Kumari et al, 2106). Hence authors should check the total ATP levels in RTS osteoblasts.*

**Response:** Based on the reviewer's suggestion, we examined ATP levels in RTS and FC osteoblasts. Consistent with the previous study (Kumari et al, J Cell Sci. 2016 Apr 1;129(7):1312-8.), a decrease of total ATP level was observed in RTS osteoblasts (Fig S4F of the revised manuscript). We suspect that elevated mitochondrial respiratory complex I gene expression to increase ATP production is an energy complementary outcome in order to rescue the lower ATP production in RTS osteoblasts. Taken together, these data demonstrate that, compared to FC osteoblasts, RTS osteoblasts upregulate OXPHOS and mitochondrial respiratory complex I -mediated ATP production at the expense of decreased glycolysis.

**4.** *Figure 5 C: complex I inhibition by IACS-010759 treatment increased cell viability in control cells but decreased in RTS patient cells. Please explain.*

**Response:** Thanks for the reviewer’s comments. We carefully re-examined our results using multiple FC and RTS osteoblasts. In our revised cell viability/ proliferation study (Fig 5D of the revised manuscript), IACS-010759 significantly inhibited RTS osteoblast growth but showed limited proliferation inhibition in FC cells.

**5.** *Figure 5: rotenone, a known inhibitor for complex I should be used in cell viability assay in parallel with IACS-010759. This will tell us whether IACS-010759 is a better candidate.*

**Response:** We studied the cell viabilities of RTS and FC osteoblasts after rotenone treatment. In comparison with IACS-010759 (Fig 5D of the revised manuscript), rotenone not only presented less therapeutic efficacies in inhibiting RTS osteoblasts but also showed a certain growth inhibitory effect on FC osteoblasts (Fig S5B of the revised manuscript). These findings imply that IACS-010759 is a better therapeutic compound to treat RTS osteosarcoma.

**6.** *Figure 5 C: decrease in cell viability should be supported by analyzing other parameters like senescence and apoptosis.*

**Response:** We appreciate the reviewer’s insightful comments. We examined senescence and apoptosis of RTS and FC osteoblasts upon IACS-010759 treatment. As shown in Fig 5E of the revised manuscript, IACS-010759 significantly induced cellular senescence of RTS osteoblasts. In contrast, IACS-010759 leads to decreased global Caspase3/7 activities in both RTS and FC osteoblasts (Fig S5D of the revised manuscript). Also, the sub-G1 population of RTS osteoblasts did not change significantly upon IACS-010759 (Fig S5E of the revised manuscript). Together, these findings suggest that induction of cellular senescence is one of the pharmacological mechanisms involved in IACS-010759 mediated RTS osteoblast growth inhibition.

**7.** *RTS patient cell lines exhibit increased reactive oxygen species (ROS) production. Authors should check the ROS levels after IACS-010759 treatment.*

**Response:** Based on the reviewer’s suggestion, we examined ROS levels in RTS and FC osteoblasts after IACS-010759 treatment. As shown in Fig S5C of the revised manuscript, RTS osteoblasts exhibited an increased ROS production compared to FC osteoblasts but IACS-010759 did not enhance ROS levels in both RTS and FC osteoblasts. This finding suggests ROS is not involved in IACS-010759 mediated RTS osteoblasts growth inhibition.

**8.** *Connections need to be discussed relating to the function of RECQL4 in mitochondria, where a number of studies have been done.*

**Response:** Thanks for the reviewer’s suggestion. Although several studies have explored RECQL4 function in mitochondria, it is still nebulous if RECQL4 pathogenic variants influence mitochondrial respiratory complex I expression and function. Here, we provided evidence to link the mitochondrial respiratory complex I to RECQL4 pathogenic variants and RTS etiology. We have discussed the role of RECQL4 in mitochondria function, integrated our findings, and provided a perspective in the Discussion (line 372-383 of the revised manuscript).

**Reviewer #3**:

**1.** *Figure 5C, seems to be a viability and not cell growth measurement. This needs to be clarified, and cell growth plots provided. In addition, it would be important for the authors to confirm reduced protein expression of some of the transcripts they described to be reduced in response to the Complex I inhibitor.*

**Response:** Thanks for the reviewer’s comments. **First**, we revised the cell growth plot of RTS and FC osteoblasts upon IACS-010759 treatment (Fig 5D of the revised manuscript). **Second**, we performed immunoblotting to confirm the downregulation of EREG (cell cycle) and STK39 (MAPK signaling) in IACS-010759-treated RTS osteoblasts (Figs 5H and 5I of the revised manuscript).
